# Research on Coverage Optimization in Wireless Sensor Networks Based on an Improved Sparrow Search Algorithm

**DOI:** 10.3390/s26134076

**Published:** 2026-06-26

**Authors:** Hong Kheam, Vakhim Leang, Chamroeun Khim, Van Nhan Vo, Sovannarith Heng

**Affiliations:** 1 Department of Information Technology Engineering, Faculty of Engineering, Royal University of Phnom Penh, Phnom Penh 120404, Cambodia; kheam.hong@rupp.edu.kh (H.K.); khim.chamroeun@rupp.edu.kh (C.K.); 2 Department of Computer Science, Faculty of Science, Royal University of Phnom Penh, Phnom Penh 120404, Cambodia; leang.vakhim@rupp.edu.kh; 3 Faculty of Information Technology, Duy Tan University, Danang 550000, Vietnam; vonhanvan@dtu.edu.vn

**Keywords:** wireless sensor network, density-aware repulsive sparrow search algorithm, coverage optimization

## Abstract

Optimal node deployment in Wireless Sensor Networks (WSNs) is crucial for maximizing monitoring coverage. However, traditional metaheuristics like the Sparrow Search Algorithm (SSA) often suffer from premature convergence and redundant clustering, creating severe coverage holes. To address this, we introduce the Density-Aware Repulsive Sparrow Search Algorithm (DAR-SSA). Integrating electrostatic principles, DAR-SSA calculates a local density-based repulsive force vector to actively disperse nodes from high-density clusters. This physics-guided approach, combined with a dynamic explorer-exploiter allocation rule, ensures a computationally efficient balance between global and local search phases. Evaluated via a probabilistic sensing model, DAR-SSA significantly outperforms standard SSA, its variants (EFSSA, EASOA), and classical algorithms (PSO, GWO). In high-density urban deployments, DAR-SSA achieves a 95.25% effective coverage rate, compared to SSA’s 76.74%. In low-density environments, coverage reaches 97.12%. Validated by Wilcoxon rank-sum tests, DAR-SSA proves to be a robust, efficient framework for mitigating spatial redundancy and maximizing WSN sensing coverage.

## 1. Introduction

### 1.1. Background

Wireless Sensor Networks (WSNs) consist of autonomous devices, known as sensor nodes, that are distributed across wide areas for specific tasks [1,2]. As wireless technology has advanced [3], WSNs have become increasingly common in fields such as target tracking [4], environmental monitoring [5], and healthcare [6]. However, due to environmental constraints, sensors are often deployed randomly. This leads to both coverage gaps and areas with redundant sensors. Therefore, it is essential to optimize sensor positions to maximize coverage, minimize blind spots, and reduce deployment costs.

Metaheuristic algorithms represent a promising approach for addressing WSN node coverage challenges [7]. These algorithms can identify near-optimal solutions efficiently, even under computational resource constraints, making them highly suitable for WSN optimization [8]. Drawing inspiration from natural phenomena, such as swarm behaviors, evolutionary processes, and physical laws, metaheuristics are widely applied in various fields [9]. Their popularity stems from their simplicity, ease of implementation, and minimal parameter requirements. Well-known examples based on physical phenomena in nature include simulated annealing (SA) [10]. Additionally, examples based on evolutionary concepts include Genetic Algorithms (GAs) [11] and Differential Evolution (DE) [12]. Furthermore, examples based on animal behavior include Particle Swarm Optimization (PSO) [13], Ant Colony Optimization (ACO) [14], the Whale Optimization Algorithm (WOA) [15], Grey Wolf Optimizer (GWO) [16], and the Salp Swarm Algorithm (SSA) [17].

### 1.2. Related Works

To address the inherent limitations of the standard Sparrow Search Algorithm (SSA) [18], the recent literature has extensively explored various enhancement strategies, although a critical review reveals distinct advantages and disadvantages across these methodologies. One prominent approach utilizes chaotic mapping techniques (e.g., CLSSA [19], SFSSA [20], and CMASSA [21]) to optimize the initialization phase. While this offers the advantage of establishing a uniform, high-quality initial distribution of sensor nodes, its critical disadvantage is a strict temporal limitation. As a “Phase-Zero” strategy, it lacks reactive spatial feedback during the terminal convergence phase, rendering it powerless to prevent redundant node clustering at the final target. Another widespread strategy is algorithmic hybridization, which merges base algorithms with other swarm intelligence models like PSO, ACO, or GWO (seen in HSSA [22], ISSA-ACO [23], SSAGWO [24], and HPSBA [25]). The primary advantage of these hybrids is their robust ability to balance global exploration with precise local exploitation, successfully improving solution accuracy in complex mathematical spaces. However, their significant disadvantage lies in their increased computational complexity and tendency to treat WSN nodes purely as abstract data points, thereby failing to address the fundamental physical geometry and spacing constraints of hardware deployment. Finally, to prevent premature convergence, contemporary variants frequently incorporate stochastic disturbance mechanisms such as Lévy flights or Cauchy mutation (e.g., AWSSA [26], ASFSSA [27], EFSSA [28], and EASOA [29]). These operators are highly advantageous for aggressively forcing stagnant agents out of local optima and maintaining population diversity. However, they are somewhat blind to their environment. In real-world WSN deployment, relying on undirected jumps can inadvertently push sensor nodes outside the target monitoring area, which can create new coverage holes. Beyond the standard SSA, a wide variety of advanced swarm intelligence and evolutionary algorithms have recently been adapted to resolve WSN coverage challenges, demonstrating the ongoing relevance of metaheuristic optimization in this domain [30,31,32,33,34,35,36]. Concurrently, the core Sparrow Search Algorithm continues to prove its high versatility, having been successfully hybridized and applied to complex engineering problems outside of spatial monitoring, such as solar photovoltaic parameter prediction [37], cloud manufacturing service composition [38], and variational modal decomposition [39]. To address the limitations of swarm intelligence, researchers frequently integrate physics-inspired mechanics and diversity strategies. For instance, distance-based repulsion and dynamic diversity evaluations successfully prevent spatial clustering in Particle Swarm Optimization [40,41,42], while the Gravitational Search and Fireworks Algorithms use adaptive spatial strategies to balance search phases [43,44,45]. Furthermore, thermodynamic concepts like simulated annealing cooling schedules [46] and the expansion of SSA into advanced predictive modeling [47] demonstrate the value of time-varying constraints. Collectively, these established physical and thermodynamic strategies directly inspire the repulsive and cooling mechanisms of our proposed DAR-SSA. Ultimately, the critical limitation shared across existing approaches is their reliance on mathematical stochasticity at the expense of deterministic spatial awareness. This fundamental gap motivates the proposed Density-Aware Repulsive Sparrow Search Algorithm (DAR-SSA), which replaces environmentally blind stochastic jumps with a physics-guided repulsive force to safely dismantle spatial node overlap and maximize effective geometric coverage. To provide a rigorous and comprehensive evaluation, this study compares the proposed DAR-SSA not only against recent SSA variants but also against these established, advanced optimization methods, including GWO and PSO.

### 1.3. Contributions

While recent research on node deployment in Wireless Sensor Networks (WSNs) has provided promising results, significant challenges remain. A primary limitation is that most studies focus on specific, isolated scenarios, failing to address algorithm performance across diverse environments. This lack of versatility makes practical application difficult. Furthermore, although the standard Sparrow Search Algorithm (SSA) and its recent variants have improved WSN optimization, they continue to struggle with multi-objective problems and are prone to premature convergence (i.e., becoming trapped in local optima). To overcome these persistent limitations, this study proposes the Density-Aware Repulsive Sparrow Search Algorithm (DAR-SSA) by integrating physics-guided stochastic principles, specifically electrostatics. This approach provides a robust and coherent framework designed to resolve the deficiencies of the standard SSA and significantly enhance node deployment coverage.

The main contributions are outlined as follows:

1. The standard danger-aware update mechanism of SSA is replaced with a novel repulsive force vector. Unlike traditional distance-penalized models that rely on classical inverse-square laws ($\frac{1}{d^{2}}$) and inherently suffer from mathematical singularities during dense initializations, DAR-SSA utilizes a Soft-Core Repulsive Potential field with a bounded exponential decay term. This ensures that agents actively and safely disperse from crowded regions, smoothly mitigating WSN node clustering without triggering spatial overflow.
2. A robust, constant-spark candidate generation method is introduced to mitigate stagnation. By assigning a fixed spark count (s) and a stable perturbation amplitude ($A_{base}$) to underperforming agents, the algorithm orchestrates an active, highly stable recovery mechanism. This approach significantly reduces the computational complexity associated with dynamic resource allocation while achieving superior convergence, particularly in unimodal search spaces.
3. The static producer–scrounger ratio (typically 80/20) is replaced by non-linear, time-dependent functions. This allows the algorithm to automatically transition from early-stage exploration to late-stage exploitation.
4. The proposed DAR-SSA is applied to the Wireless Sensor Network (WSN) coverage optimization problem using a realistic probabilistic sensing model rather than a simplified binary model, achieving a robust development scheme that maximizes effective coverage area.
5. DAR-SSA’s performance is validated by comparing it against the traditional SSA, two SSA variants, and advanced optimization methods (PSO and GWO). Experiments are conducted on standard benchmark functions and wireless sensor node coverage deployment to verify its robustness and effectiveness.
6. To ensure our proposed algorithm performs well for both standard benchmark functions and WSN coverage, we apply Wilcoxon test results with algorithm ranking and significant comparisons.

### 1.4. Paper Organization

The remainder of this paper is organized as follows: Section 2 establishes the mathematical models for WSN coverage. Section 3 provides a theoretical overview of the standard Sparrow Search Algorithm (SSA). Section 4 elaborates on the proposed DAR-SSA and details its implementation for optimizing WSN node deployment. Section 5 presents a comprehensive experimental analysis to validate the effectiveness of the proposed algorithm. Finally, Section 6 summarizes the entire paper and outlines future research directions.

## 2. System Model

The node coverage model represents a fundamental challenge in Wireless Sensor Networks (WSNs), requiring the strategic determination of node placement and quantity. The primary objective is to maximize the effective coverage area with a certain number of nodes or to ensure complete coverage of a designated target region. Specifically, the WSN node coverage optimization problem addresses scenarios where each sensor operates within a defined core sensing radius, a sensing radius, and a denoted extended radius at its deployed location. Within this monitoring area, individual sensors are constrained to perform sensing and exploration strictly within the limits of the core sensing, sensing, and denoted extended radii [48].

Consequently, node deployment must strictly adhere to constraints regarding the core, sensing, and extended sensing radii to minimize redundant overlaps and ensure optimal monitoring coverage across the target area. The scope of this study is strictly confined to spatial coverage optimization. Our objective function focuses solely on maximizing the effective geometric sensing area and mitigating node redundancy. Consequently, complex network connectivity parameters such as communication radius, base station reachability, connected graph constraints, and explicit routing paths are not modeled in this study. We treat data routing and network connectivity as a separate protocol layer that falls outside the scope of this geometric deployment optimization.

**Figure 1:**
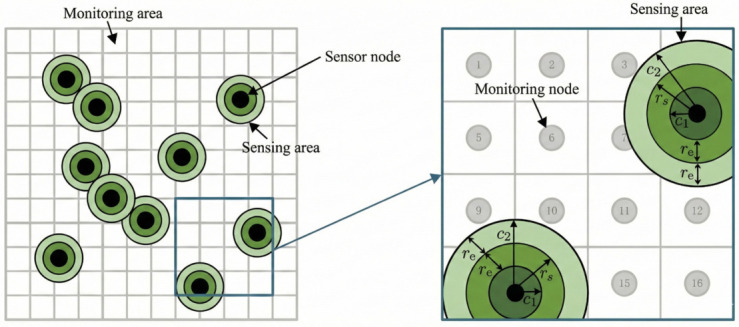
WSN monitoring models.

The WSN monitoring models are illustrated in Figure 1. We consider a two-dimensional monitoring area of $L=H\times W\left(m^{2}\right)$. Within this area, N sensor nodes are randomly initialized. Let the set of nodes be denoted as M, represented as $M\;=\;\{x_{i}\;|\;i=1,2,\cdot \cdot \cdot ,N\}$. The coordinates of $M_{i}$ are ${\{(x}_{i},\;y_{i})\;|\;i=1,2,\cdot \cdot \cdot ,N\}$. The sensing capabilities of each sensor node are defined by a circular range in the sensing area.

To facilitate the computation, the rectangular monitoring area of the deployed network is divided into H×W equal-area grids. The set of target grid points to be monitored, denoted as $G\;=\;\{x_{j}\;|\;j=1,2,\cdot \cdot \cdot ,L\;=\;H\times W\}$, is located at the center of each grid. The coordinates of $G_{j}$ are. The Euclidean distance between the sensor node $M_{i}$ and the target grid point $G_{j}$ is defined as:

(1) $$d\left(M_{i},G_{j}\right)=\sqrt{{\left(x_{i}-x_{j}\right)}^{2}+{\left(y_{i}-y_{j}\right)}^{2}}$$

To accurately characterize sensing performance within the target region, this study adopts a probabilistic sensing model. This approach is effective for uncertainties caused by environmental noise and signal interference. Unlike the traditional binary (Boolean) model, which assumes a target is either perfectly detected or completely undetected based on a fixed radius, the probabilistic model captures the realistic gradual decay in sensing accuracy as the distance from the sensor increases. Accordingly, the probability of a sensor node detecting a target is defined as follows:

(2) $$P\left(M_{i},G_{j}\right)\;=\;\{\begin{array}{c} 1\;\;\;\;\;\;\;\;\;\;\;\;\;\;\;\;\;\;\;\;\;\;\;ifd\left(M_{i},G_{j}\right)\leq c_{1}\\ e^{-\lambda \alpha^{\beta }}\;\;\;if.c_{1}<d\left(M_{i},G_{j}\right)<c_{2}\\ 0\;\;\;\;\;\;\;\;\;\;\;\;\;\;\;\;\;\;\;\;\;\;\;ifd\left(M_{i},G_{j}\right)\geq c_{2} \end{array}$$

where $c_{1}=r_{s}-r_{e}$ and $c_{2}=r_{s}+r_{e}$ represent the boundaries of the sensing capability. $c_{1}$ defines the core sensing radius, within which target detection is considered certain, while $r_{s}$ is the sensing radius of the sensor nodes that have been defined by the hardware, within which target detection is considered nearly certain based on the errors. Conversely, the region extends beyond $c_{1}$ up to $c_{2}$, representing the uncertain sensing zone, where the detection probability decays exponentially. The term $\alpha =d\left(M_{i},G_{j}\right)-c_{1}$ quantifies the distance extending beyond the reliable core radius. The parameters λ, β, and γ control the rate and shape of this exponential decay, enabling the model to adapt to varying sensor characteristics and environmental conditions.

If a target location is monitored by multiple sensor nodes, the total sensing probability increases due to cooperative detection. We define the joint probability P to represent the likelihood that the target is sensed by the collective network. The joint probability P is defined as follows:

(3) $$P\left(M,G_{j}\right)=1-\prod_{i=1}^{N}\left[1-P\left(M_{i},G_{j}\right)\;\right]\;$$

where $P\left(M,G_{j}\right)$ is the total sensing probability, N represents the total number of sensor nodes, and M denotes the set containing all deployed sensors. The primary objective of the WSN coverage optimization problem is to identify an effective node deployment strategy that maximizes coverage. The coverage rate of a WSN can be characterized as the ratio of the number of targets covered by the sensor set to the total number of targets in the area, expressed as:

(4) $$Cov=\frac{\sum_{j=1}^{L}P\left(M,G_{j}\right)}{H\times W}$$

where *Cov* represents the coverage rate of the area, which is used as an objective function in the WSN coverage optimization.

## 3. Sparrow Search Algorithm

### 3.1. Overview of the Sparrow Search Algorithm

In 2020, the Sparrow Search Algorithm (SSA) was introduced as a novel metaheuristic technique inspired by the collective foraging dynamics and vigilance behaviors of sparrows [18]. In this model, the sparrow population is conceptually divided into two groups: producers (also known as discoverers) and followers (or joiners). Producers are typically characterized by high energy reserves represented by superior fitness values and are responsible for exploring the search space to identify food sources and guide the population’s movement. Followers, in turn, utilize the positional information provided by producers to exploit these resource areas. Furthermore, SSA integrates a vital early warning mechanism: a subset of the population is tasked with vigilance, enabling the flock to detect potential threats and initiate anti-predatory behaviors to avoid danger.

In the simulation experiment, virtual sparrows are used to find food. The sparrows’ positions can be represented by the following matrix:

(5) $$X=\left[\begin{array}{cc} \begin{array}{ccc} x_{1,1} & x_{1,2} & \ldots \end{array} & \begin{array}{cc} \ldots & x_{1,d} \end{array}\\ \begin{array}{ccc} \begin{array}{c} x_{2,1}\\ \begin{array}{c} \vdots \\ x_{n,1} \end{array} \end{array} & \begin{array}{c} x_{2,2}\\ \begin{array}{c} \vdots \\ x_{n,2} \end{array} \end{array} & \begin{array}{c} \ldots \\ \begin{array}{c} \vdots \\ \ldots \end{array} \end{array} \end{array} & \begin{array}{cc} \begin{array}{c} \ldots \\ \begin{array}{c} \vdots \\ \ldots \end{array} \end{array} & \begin{array}{c} x_{2,d}\\ \begin{array}{c} \vdots \\ x_{n,d} \end{array} \end{array} \end{array} \end{array}\right]$$

where *n* is the number of sparrows and *d* is the dimension of the variables to be optimized. Then, the fitness value of all sparrows can be expressed as the following vector:

(6) $$F_{X}=\left[\begin{array}{cc} \begin{array}{ccc} {f([x}_{1,1} & x_{1,2} & \ldots \end{array} & \begin{array}{cc} \ldots & x_{1,d} \end{array}])\\ \begin{array}{ccc} \begin{array}{c} {f([x}_{2,1}\\ \begin{array}{c} \vdots \\ {f([x}_{n,1} \end{array} \end{array} & \begin{array}{c} x_{2,2}\\ \begin{array}{c} \vdots \\ x_{n,2} \end{array} \end{array} & \begin{array}{c} \ldots \\ \begin{array}{c} \vdots \\ \ldots \end{array} \end{array} \end{array} & \begin{array}{cc} \begin{array}{c} \ldots \\ \begin{array}{c} \vdots \\ \ldots \end{array} \end{array} & \begin{array}{c} x_{2,d}])\\ \begin{array}{c} \vdots \\ x_{n,d}]) \end{array} \end{array} \end{array} \end{array}\right]$$

The value of each row in $F_{X}$ represents the fitness value of an individual.

### 3.2. Update Producer

In SSA, producers with higher fitness values lead the foraging process and are granted priority in food acquisition. Due to their role in guiding swarms, the producers are responsible for exploring a broader range than the followers. Consequently, each producer’s position is updated according to the equation below:

(7) $$X_{i,\;j}^{t+1}=\{\begin{array}{c} X_{i,\;j}^{t}\cdot exp(\frac{-i}{\alpha \;\cdot \;{iter}_{max}})\;\;\;\;if\;R_{2}<ST\\ X_{i,\;j}^{t}+Q\cdot L.\;\;\;\;\;\;\;\;\;\;\;\;\;\;\;\;\;\;\;if\;R_{2}\geq ST \end{array}$$

where t denotes the current iteration, j=1,2,…,d. The term $X_{i,j}^{t}$ represents the position of the i-th sparrow in the j-th dimension at iteration t. The constant ${iter}_{max}$ indicates the maximum number of iterations. αϵ(0,1] is a random number, while $R_{2}\epsilon \left[0,1\right]$ and STϵ[0.5,1.0] represent the alarm value and the safety threshold, respectively. Q is a random number following a normal distribution. L denotes a 1×d matrix in which each element is 1.

$R_{2}<ST$ implies that no predators are detected, and the producer enters the wide search mode. $R_{2}\geq ST$ implies that a predator is detected. In response, the sparrows must quickly fly to other safe areas to avoid the threat.

### 3.3. Update Follower

The behavior of followers (scroungers) is defined in (8) and (9). As previously discussed, followers continuously monitor producers. Upon detecting that a producer has identified a high-quality food source, followers immediately leave their current positions to compete for the resource. If a follower successfully competes for the food, it occupies the producer’s location. Otherwise, it executes the movement strategy defined in (9). The specific position update formula for the scroungers is described as follows:

(8) $$X_{i,j}^{t+1}=\{\begin{array}{c} Q\cdot exp(\frac{X_{worst}^{t}\;-{\;X}_{i,j}^{t}}{i^{2}})\;\;\;\;\;\;\;\;\;\;\;\;\;\;\;\;\;\;\;if\;i\;>\;n/2\\ X_{best}^{t+1}+\left|X_{i,\;j}^{t}-X_{best}^{t+1}\right|\cdot A^{+}\cdot L\;\;\;otherwise \end{array}$$

where $X_{best}^{t+1}$ represents the global best position in the current iteration result, serving as the elite solution. $X_{worst}^{t}$ denotes the current global worst location in the current iteration result. A represents a 1×d matrix in which each element inside is randomly assigned 1 or −1, and $A^{+}=A^{T}{\left(AA^{T}\right)}^{-1}$. The condition i > n/2 indicates that the i-th scrounger with the worst fitness value is most likely to be starving, necessitating migration to other areas to forage for energy.

### 3.4. Update Danger Awareness of Sparrows

In the original SSA formulation, it is assumed that a specific subset of sparrows, typically accounting for 10% to 20% of the total population, is aware of potential danger. These individuals are randomly selected in each iteration to perform anti-predatory behaviors. The position update formula for these vigilant sparrows is defined as:

(9) $$X_{i,j}^{t+1}=\{\begin{array}{c} X_{best}^{t}+\beta \cdot \left|X_{i,\;j}^{t}-X_{best}^{t}\right|\;if\;f_{i}\;>\;f_{g}\\ X_{i,j}^{t}+K\cdot (\frac{\left|X_{i,\;j}^{t}-X_{worst}^{t}\right|}{(f_{i}-f_{w})+\varepsilon })\;\;\;\;\;\;if\;f_{i}\;=\;f_{g} \end{array}$$

where $X_{best}^{t}$ denotes the current global optimal position in the current iteration. The parameter β serves as a step size control coefficient, following a standard normal distribution with a mean of 0 and a variance of 1. K∈[−1.1] is a random variable that determines the direction of movement. $f_{i}$ represents the fitness value of the current sparrow, while $f_{g}$ and $f_{w}$ correspond to the current global best and worst fitness values, respectively. ε is a small constant introduced to prevent zero-division errors. In terms of behavioral logic, $f_{i}>f_{g}$ indicates that the sparrow is located at the periphery of the group. Since $X_{best}^{t}$ represents the safe centers of the population in the current iteration of the population, the sparrow moves to $X_{best}^{t}$ to seek safety, while $f_{i}=f_{g}$ implies that the sparrow is already in the central (optimal) position but has detected a threat. Consequently, it must adjust its position to evade predation. Here, K controls both the direction of movement and the step length required to move closer to other individuals for protection.

## 4. Proposed Density-Aware Repulsive Sparrow Search Algorithm

### 4.1. Non-Linear Adaptive State Allocation

The fixed global explorer–local exploiter ratio in the original SSA limits performance. To improve efficiency, the population structure should evolve, prioritizing global explorers early for global exploration and shifting to local exploiters later for local exploitation. The proposed adaptive mechanism for calculating this dynamic ratio is described below:

(10) $$R(t)=R_{end}+(R_{start}-R_{end})\cdot (1-{\left(\frac{t}{T_{max}}\right)}^{\lambda })$$

The global explorer ratio R(t) is defined to be controlled by a non-linear function at iteration t. $R_{start}$ is the initial global explorer ratio, set to be 80% of the population at iteration *t* = 0. $R_{end}$ is the final global explorer ratio, set to be 20% of the population at iteration $t=T_{max}$. The transition exponent is λ=2 for a non-linear or convex transition.

**Figure 2:**
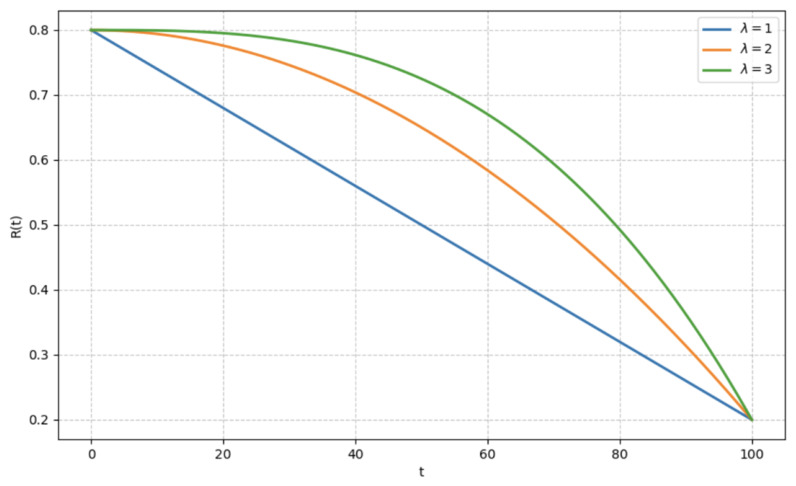
Adaptive convergence state controller.

Figure 2 compares different decay rates. With λ=1, the linear drop is too uniform and lacks adaptability when it reaches the end. λ=3 is often too aggressive; it waits too long to drop and causes a sudden crash at the end. In contrast, λ=2 provides a natural curve. It introduces non-linearity with a convex curve that balances the transition speed, smoothing the shift from exploration (high global explorer count) to exploitation (low global explorer count).

At the beginning of each iteration t, roles are re-assigned as:

(11) $$N_{p}\left(t\right)=round(N\left(t\right)\cdot R\left(t\right))$$

The population N(t) is sorted by fitness at iteration t, and the top $N_{p}\left(t\right)$ individuals are assigned as the global explorer role, where $N_{p}\left(t\right)$ is determined by the adaptive ratio described previously.

### 4.2. Density and Repulsion Calculation (Physics-Based)

The distances between a search agent and its neighboring search agents are defined as:

(12) $$d_{i\;j}=\left\|X_{i}-X_{j}\right\|$$

where $X_{i}$ represents the position of search agent i and $X_{j}$ represents the position of search agent j. $d_{i\;j}$ represents the distance between search agents i and j. Although SSA’s group foraging strategy performs well on benchmark functions, it causes node clustering in coverage applications, resulting in significant overlap. To address the premature convergence and physical clustering of agents, the crowding threshold $D_{th}$ is introduced to define the minimum optimal distance between two nodes. While a standard Boolean disk model might define this strictly by the nominal sensing radius, our probabilistic sensing model requires a boundary condition that accounts for the exponential decay of detection probability. The ideal spatial separation between two probabilistic sensors occurs precisely when the absolute certainty zone (the core radius, $c_{1}=r_{s}-r_{e}$) of one sensor meets the absolute uncertainty boundary (the extended edge, $c_{2}=r_{s}+r_{e}$) of a neighboring sensor. By explicitly tying the density threshold to this probabilistic interaction, the highly effective boundary condition is formulated as:

(13) $$D_{th}=c_{1}+c_{2}={(r}_{s}-r_{e})+(r_{s}+r_{e})=2\cdot r_{s}$$

Thus, while the threshold computationally simplifies to $2\cdot r_{s}$, its theoretical foundation is rigorously grounded in the interaction between the probabilistic sensing bands, ensuring the density evaluation physically aligns with the nodes’ true sensing decay zones.

#### Engineering Imperative by Translating Spatial Overlap to Coverage Efficiency

In the physical deployment of a Wireless Sensor Network, the total monitoring capacity is strictly constrained by the finite number of available sensor nodes. To achieve optimal performance, each node must contribute maximally to the overall spatial coverage of the target area. When traditional swarm algorithms (such as the standard SSA or WOA) optimize node placement, their inherent tendency to aggressively exploit local optima causes agents to cluster tightly together. When nodes overlap geometrically, their sensing radii intersect, meaning multiple sensors redundantly monitor the exact same spatial zone.

Consequently, this severe spatial overlap wastes the fundamental sensing capability of the network. By squandering their coverage potential on an already-monitored area, fewer nodes remain available to deploy into unexplored or entirely unmonitored regions. The network suffers from acute spatial inefficiency, where high-density clusters provide zero additional geometric benefit while leaving other zones completely exposed. This localized redundancy leads directly to the creation of structural coverage holes, critical geographical areas that remain permanent blind spots simply because the nodes that should have covered them are instead trapped in redundant clusters.

To resolve this spatial inefficiency, introducing a deterministic repulsive force is an engineering imperative. Rather than serving as a mechanism to prevent node redundancy, this repulsive force actively translates spatial overlap into a directional vector that pushes clustered nodes apart. By forcing overlapping sensors away from one another, their sensing radii are redistributed across a wider, distinct geometric area. This ensures that every node is pushed toward unexplored terrain, systematically eliminating spatial redundancy and maximizing the effective global coverage rate of the network.

**Figure 3:**
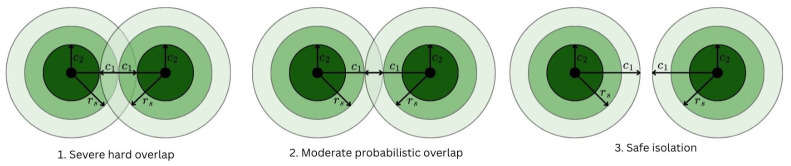
Boundary conditions for the crowding threshold relative to the sensor radius.

Consequently, Figure 3 illustrates how the density kernel functions evaluate the spatial relationships between nodes through the lens of these probabilistic bands, categorizing the coverage overlap into three distinct operational states as follows:

1. $d_{i\;j}<2\cdot c_{1}$ (Severe hard overlap): At this close proximity, the absolute certainty cores of adjacent sensors directly intersect. The network suffers from severe spatial redundancy and wasted energy. To mitigate this, the algorithm triggers a maximum repulsive force, rapidly dispersing the heavily overlapping nodes to preserve network longevity.
2. ${2\cdot c}_{1}\leq d_{i\;j}\leq 2\cdot c_{2}$ (Moderate probabilistic overlap): In this transitional state, the distances fall within the sensor decay zones. Sensing still overlaps probabilistically, but certainty decreases as distance increases. This zone triggers a scaled, adaptive repulsive weight that smoothly guides nodes toward optimal separation without causing erratic scattering.
3. $d_{i\;j}>2\cdot c_{2}$ (Safe isolation): Once the distance strictly exceeds the combined uncertainty boundaries, the nodes operate in an independent zone without sensing interference. Because no probabilistic overlap exists here, the repulsive weight becomes zero, allowing the agents to dedicate their movement entirely to the primary exploration and exploitation objectives.

To accurately evaluate the spatial distribution of the search agent, it is necessary to move beyond the simple connectivity metrics. Traditional approaches often rely on a simple count of neighbors to determine density. However, this binary approach fails to capture the severity of the sensing overlap, as it treats a neighbor at the periphery of the sensing range identically to one at the exact same coordinates.

To address this limitation, a local density ${(\rho }_{i})$ metric is introduced. Rather than a discrete count, $\rho_{i}$ is defined as a weighted sum of proximities. This metric acts as a continuous crowdedness score, differentiating between dense clusters, where search agents are critically close (high overlap), and loose clusters, where search agents are spaced enough. To model the severity of the overlap, this study introduces the equation below:

(14) $$\rho_{i}=\sum_{j}^{N_{i}}K(d_{i\;j})$$

(15) $$K(d_{i\;j})=\{\begin{array}{c} 1-{\left(\frac{d_{i\;j}}{D_{th}}\right)}^{2}\;\;\;\;if\;0<d_{i\;j}<D_{th}\\ 0\;\;\;\;\;\;\;\;\;\;\;\;\;\;\;\;\;\;\;\;\;\;\;\;\;\;\;\;\;\;\;\;\;\;otherwise \end{array}$$

**Figure 4:**
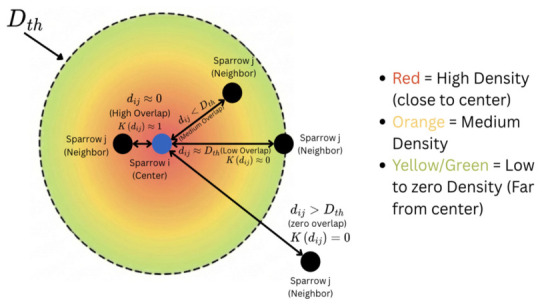
Local density of each search agent (sparrow).

$N_{i}$ is the set of the neighbors within distance $D_{th}$. Figure 4 illustrates how search agent i receives density from their neighbors’ search agent j.

**Figure 5:**
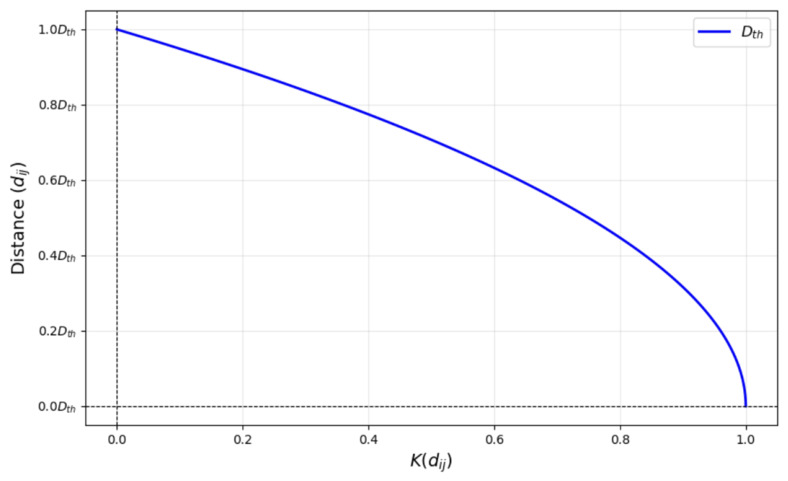
Total local density for each search agent.

Figure 4 and Figure 5, and Equation (14) represent the kernel functions established as a smooth, non-linear density gradient that operates on these boundary principles. When nodes are perfectly overlapping ($d_{i\;j}\to 0$), the function assigns the highest possible weight. This indicates that for very small distances, the weight remains robustly close to 1.0. When the distance hits the threshold ($d_{i\;j}\to D_{th}$), the function forces the weight to zero, ensuring that any node outside this radius contributes absolutely nothing to the density calculation. As the distance decreases further, the curve bends more sharply to the right. This indicates how the distance penalty accelerates, leading search agents approaching the threshold to lose their influence weight much faster than close search agents. To maintain population diversity and prevent premature convergence, a repulsive force is applied to search agent i based on the proximity of its neighbors.

**Figure 6:**
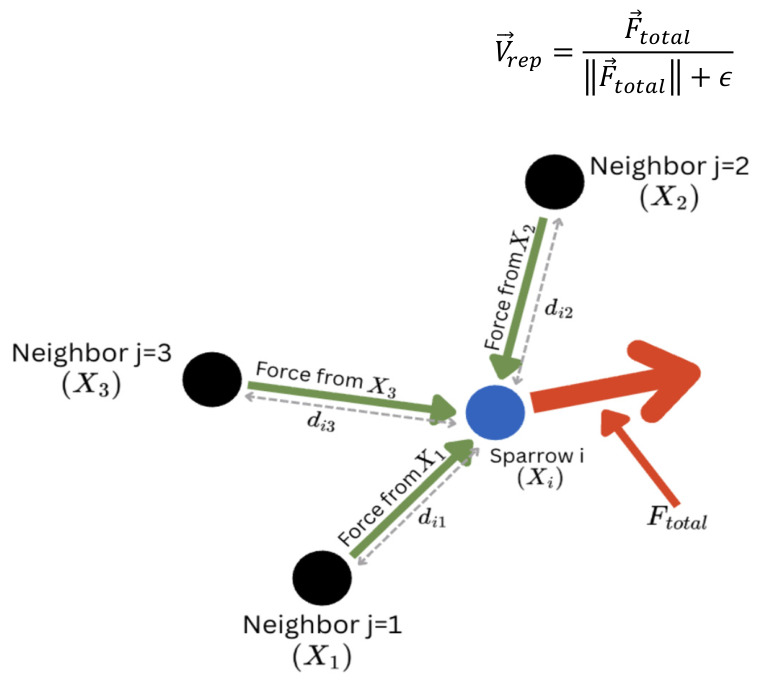
Total repulsive force for a search agent (sparrow).

As shown in Figure 6, the total repulsive force $F_{total}$ is calculated as the summation of interactions with $N_{i}$ neighboring search agents:

(16) $${\vec{F}}_{total}=\sum_{j\in N_{i}}\frac{{\vec{X}}_{i}-{\vec{X}}_{j}}{d_{i\;j}}\cdot exp(-\frac{d_{i\;j}}{R_{s}})$$

where $X_{i}$ and $X_{j}$ represent the position vectors of the current search agent and neighbor j, respectively, $d_{i\;j}$ is the Euclidean distance between them, and $R_{s}$ is a scaling constant. The final repulsive vector is obtained by normalizing $F_{total}$ to ensure consistent magnitude handling:

(17) $${\vec{V}}_{rep}=\frac{{\vec{F}}_{total}}{\left\|{\vec{F}}_{total}\right\|+\epsilon }$$

A critical feature of this formulation and a primary distinction from traditional repulsive swarm intelligence models like distance-penalized Particle Swarm Optimization (PSO) is the implementation of a Soft-Core Repulsive Potential field. Traditional algorithms frequently rely on variations of Coulomb’s law or classical inverse-square models ($1/d^{2}$). The fundamental mathematical flaw in applying inverse-square laws to computational WSN optimization is the creation of singularities. As the inter-node distance ($d_{i\;j}$) approaches zero, a state that frequently occurs during random initializations or dense spatial clustering, the calculated repulsive force approaches infinity. In computational optimization, this singularity leads to severe numerical instability, floating-point overflow, and the aggressive, uncontrolled ejection of search agents far beyond the functional boundaries of the search space.

To resolve this, DAR-SSA replaces the dangerous inverse-square singularity with an exponentially decaying term, $exp\left(-d_{i\;j}/R_{s}\right)$. By utilizing this function, the algorithm acts as a bounded soft-core potential. As the distance between nodes approaches zero ($d_{i\;j}\to 0$), the repulsive force does not reach infinity but instead smoothly plateaus at a finite maximum constant. This ensures absolute gradient stability even during the densest network initializations. This precise coupling of soft-core physical repulsion with thermodynamic resource allocation represents a novel paradigm specifically engineered to safely dismantle terminal-phase WSN node clustering.

### 4.3. Density-Aware Candidate Generation (Hybrid Update)

This section defines the core movement mechanics of the algorithm. The candidate generation process is governed by a hybrid update rule that balances environmental pressure against temporal cooling. First, we establish a global convergence constraint using a Time-Varying Decay (TVRD) coefficient, denoted as δ(t). This mechanism functions as a system cooling schedule, similar to the temperature reduction in simulated annealing:

(18) $$\delta \left(t\right)={\left(1-\frac{t}{T_{max}}\right)}^{\gamma }$$

where δ(t) serves as a global damper. At t=0, the system has maximum energy (δ(t)=1), allowing for unrestricted movement. As $t\to T_{max}$, δ(t)→0, effectively freezing the system to ensure stability and precision in the final iterations. To facilitate a robust balance between global exploration and local exploitation, the candidate generation process utilizes an Adaptive Amplitude ($A_{i}$) mechanism. This mechanism modulates the step size of each search agent based on both the current iteration number and the local population density. This factor acts as a scaling coefficient that decreases from 1 to 0. The amplitude $A_{i}$ is computed as the product of baseline potential, density factor, and temporal decay:

(19) $$A_{i}=A_{base}\cdot (1+\Gamma \cdot \rho_{i})\cdot \delta \left(t\right)$$

The parameter Γ is introduced as a repulsion gain coefficient. It modulates the sensitivity of the search radius $A_{i}$ to the local population density $\rho_{i}$. By scaling the density term, Γ allows the algorithm to be tuned for different optimization landscapes. A larger Γ induces stronger repulsive forces to escape deep local optima, while a smaller Γ preserves fine-grained exploitation capabilities.

The movement trajectory is defined by a hybrid vector, $d_{hybrid}$, which synthesizes probabilistic exploration with density-aware environmental evasion. The direction is computed via a biased stochastic vector addition as follows:

(20) $${\vec{d}}_{hybrid}=(1-\omega ){\vec{r}}_{rand}+\omega {\vec{V}}_{rep}$$

where $r_{rand}$ is a normalized random vector drawn from a uniform distribution, providing the fundamental stochasticity required for global search and exploration. $V_{rep}$ is the physics-inspired repulsion vector calculated from the exact spatial geometry and local densities of the neighborhood. This vector provides a directional bias away from population clusters. ωϵ[0,1] is the weighting factor that regulates the influence of this physical bias. By superimposing the calculated repulsive bias ($V_{rep}$) onto the stochastic random vector ($r_{rand}$), the algorithm implements a physics-guided biased random walk, achieving environmental evasion without eliminating the probabilistic exploration essential to swarm intelligence. This hybrid approach ensures that while the search agent moves stochastically, its overall trajectory drifts away from over-densified regions, thereby reducing the probability of collision and redundant searching.

To generate the final candidate solution, the algorithm applies the computed movement vector to the current position. The update mechanism incorporates a dimensional scaling factor to map the normalized algorithmic parameters onto the specific constraints of the search space:

(21) $$X_{new}=X_{old}+A_{i}\cdot {\vec{d}}_{hybrid}\cdot (UB-LB)$$

The components $A_{i}$ (amplitude) and $d_{hybrid}$ (direction) are inherently unitless relative to the problem constraints. To ensure valid exploration, the term (UB−LB) is applied as a dimensional scalar. This operation denormalizes the step vector, converting the relative jump size into absolute physical units appropriate for the solution space.

To ensure the convergence of the algorithm, a greedy selection strategy is applied to the candidate solutions. While the hybrid movement vector allows search agents to explore and avoid crowding, it is stochastic and does not guarantee an immediate improvement in fitness. Therefore, the update rule evaluates the quality of the candidate position $X_{new}$ against the current position $X_{i}^{t}$. The search agent moves to the new location only if the fitness improves; otherwise, it retains its previous position:

(22) $$X_{i}^{t+1}=\{\begin{array}{c} X_{new}\;\;\;\;if\;f(X_{new})<f(X_{i}^{t})\\ X_{i}^{t}\;\;\;\;\;\;\;\;\;\;\;\;\;\;\;\;\;\;\;\;\;\;\;otherwise \end{array}$$

### 4.4. Parameter Sensitivity and Contextual Scaling

A critical feature of the proposed algorithm is the adaptive definition of the sensing radius $R_{s}$. In engineering applications like WSN coverage optimization, $R_{s}$ is dictated by the physical properties of the sensor. However, in unconstrained numerical optimization, utilizing fixed absolute values for $R_{s}$ would be ineffective due to variation in domain sizes across different benchmark functions.

**Figure 7:**
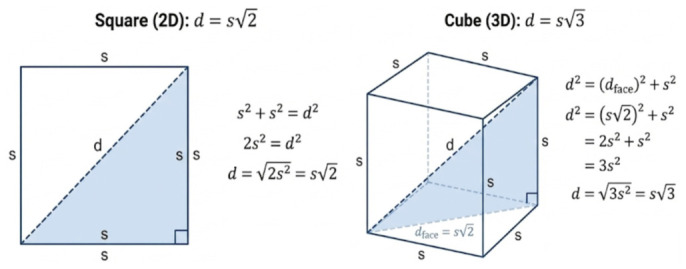
Diagonal length.

To address this, a dimension-aware scaling mechanism is introduced. As illustrated by the geometric principles in Figure 7, the diagonal length of the search space increases with dimensionality. Consequently, for standard test functions, $R_{s}$ is dynamically computed to maintain scale invariance:

(23) $$R_{s}=C_{scale}\cdot \sqrt{dim}\cdot (UB-LB)$$

This formulation ensures that the interaction range scales linearly with the diagonal size of the hypercube search space. Consequently, the pressure exerted by neighboring search agents remains consistent, whether the algorithm is solving a 2D problem or a 100D problem. We set $C_{scale}=5\%$ of a total diagonal length within the hypercube search space to balance local repulsive influence with global separations.

**Figure 8:**
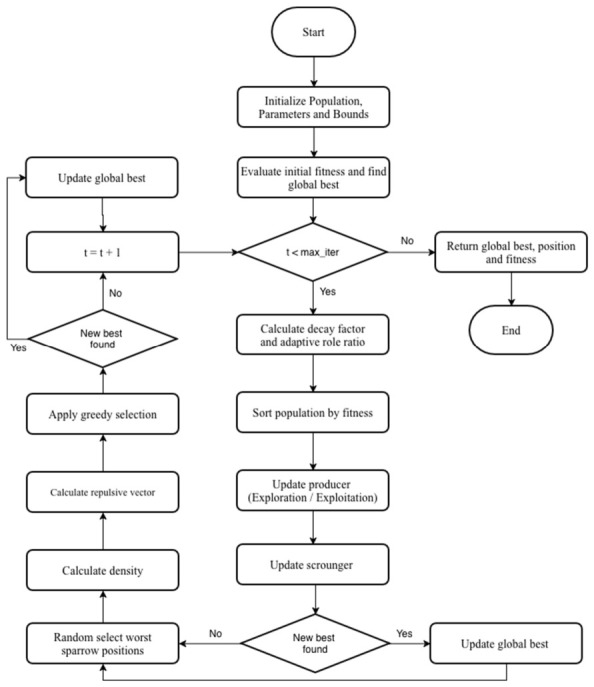
Flowchart of DAR-SSA.

The complete computational procedure for the proposed DAR-SSA is outlined in Algorithm 1, and its corresponding step-by-step execution workflow is illustrated in Figure 8.
**Algorithm 1.** Pseudocode for DAR-SSA.**Input**:
Parameter: Population size (N), Dimension (dim), Bounds (LB, UB), Max Iterations ($T_{max}$) SSA Constants: ST (Safety threshold), PD (global explorer number), SD (search agent perceive danger) DAR-SSA Constants: s (Spark counts), $A_{base}$(amplitudes), γ(decay) **Output**:
Global best positing ($X_{best}$), fitness ($F_{best}$) Initialize population X with random positions between LB and UBCalculate the fitness for all individuals $F_{i}=objFunc\left(X_{i}\right)$ for i = 1…NIdentify the global best solution $X_{best}$ and $F_{best}$While (*t* < $T_{max}$):          Using Equation (18) to calculate decay factor          Using Equation (10) to calculate adaptive role          Sort population X and their fitness F from the best to worst          for *i* = 1 to $N_{p}$                    Using Equation (7) to update search agent’s location          Clamp $X_{i}$ within bounds LB, UB and recalculate fitness $F_{i}$          for *i* = $N_{p}$ + 1 to *N*                    Using Equation (8) to update search agent’s location          Clamp $X_{i}$ within bounds LB, UB and recalculate fitness $F_{i}$          Find the current best in population. If better than $X_{best}$, update $X_{best}$ and $F_{best}$          Select SD indices randomly from the population.          for each selected index k in SD                    Using Equation (12) for pairwise distance                    Using Equations (13)–(17) to calculate density and repulsive force                    Initialize local_best_spark                    for *j* = 1 to S (spark count)                              Using Equation (20) to generate the hybrid direction                              Using Equation (21) to displacement the position                              Clamp $X_{new}$ and evaluate $F_{new}$                              Update local_best_spark if $F_{new}$ is better                    Using Equation (22) to apply greedy selection          *t* = *t* + 1return $X_{best}$ and $F_{best}$

## 5. Simulation Experiments and Analysis

Twenty-one benchmark functions are used to test the proposed Density-Aware Repulsive Sparrow Search Algorithm, which are listed in Table 1. There are two categories of benchmark functions. F1–F11 are unimodal functions, which mark the category as U. F12–F21 are multimodal functions, which mark the category as M.

### 5.1. Standard Test Function Experiments

To evaluate the performance of the proposed DAR-SSA in solving numerical optimization problems, it is benchmarked against the original SSA [18]; two variants, namely, EFSSA [28] and EASOA [29]; and two advanced classical optimization algorithms (PSO [13] and GWO [16]). The parameter settings for these comparative algorithms are detailed in Table 2. All experiments were conducted using a Python 3.11.5 environment on a system with an Apple M1 chip and 16 GB of RAM.

#### 5.1.1. Comparison Algorithm Parameter Settings

To ensure the reasonableness and fairness of the comparison, the number of test functions in the numerical optimization experiment is set to 30, the population size is set to 100, and the maximum number of iterations is set to 500.

#### 5.1.2. Comparison Results: DAR-SSA vs. Other Algorithms

For the same test functions, each algorithm is independently run 30 times. The mean (mean), standard deviation (Std), best result (best), and worst result (worst) are calculated according to the statistical value. Table 3 shows the comparison results of three algorithms with dim = 30. The Wilcoxon rank-sum test results calculated at a significance level of 0.05 are also listed in Table 3. The second-to-last row indicates the number of successes (+), failures (−), and approximations (≈) of the compared algorithms with respect to DAR-SSA. The last row shows the rank of the compared algorithms. The results show that for functions F1, F2, F6, F7, F8, F9, F10, F11, F12, F14, F15, and F17, both the proposed DAR-SSA and variant EFSSA reach the best average performance. For functions F3 and F16, the traditional SSA performs the best. For function F11, EFSSA and the proposed algorithm DAR-SSA has the best average performance. For function F5, the traditional SSA, variant EFSSA, and the proposed algorithm DAR-SSA have the best average performance at the greatest value. For function F19, traditional SSA, variant EFSSA, PSO, and the proposed algorithm DAR-SSA have the best average performance at the greatest value. For function F21, variant EFSSA, GWO, and the proposed DAR-SSA have the best average performance at the greatest value. For function F20, the average of variant EFSSA outperforms the other algorithms. For functions F4, F13, and F18, the average of DAR-SSA outperforms the other algorithms.

**Figure 9:**
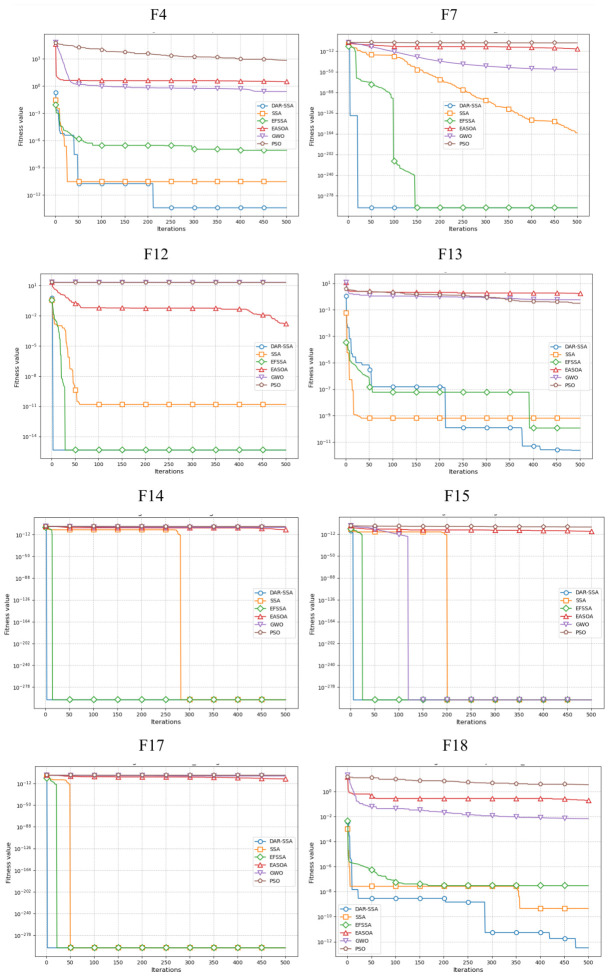
Convergence curves of the compared algorithms on selected standard benchmark functions.

The convergence performance of the proposed DAR-SSA alongside the comparative algorithms for selected benchmark functions is visualized in Figure 9. Furthermore, to validate the statistical significance of the results, Wilcoxon Signed-Rank tests were performed, as shown in Table 4. The analysis compares DAR-SSA against SSA, EFSSA, and EASOA. All resulting *p*-values (0.0056, 0.0232, 0.0000298, 0.0000443, and 0.000297, respectively) are less than 0.05. This indicates a significant performance difference, statistically confirming DAR-SSA’s superiority over the competing algorithms.

### 5.2. Component Ablation Analysis

To rigorously estimate the significance of the individual components introduced in the Density-Aware Repulsive Sparrow Search Algorithm (DAR-SSA), a component ablation study was conducted. An ablation study systematically disables distinct mechanical enhancements to isolate and quantify their specific contributions to the algorithm’s overall performance.

To manage spatial coverage overhead while ensuring a comprehensive evaluation, the analysis was executed on a representative subset of the experimental framework, the primary high-density WSN coverage configuration (Experiment 1), to validate physical applicability.

The methodology defines four specific algorithmic variants for this analysis.

1. Full DAR-SSA: The complete proposed framework containing all physical and thermodynamic enhancements.
2. DAR-SSA-NA (No Adaptation): Disables the time-varying non-linear state allocation rule, forcing the algorithm to rely on the standard, static 80/20 discoverer–follower ratio throughout all iterations.
3. DAR-SSA-NR (No Repulsion): Completely removes the density-aware repulsive vector ($V_{rep}$). This variant relies purely on the standard danger-aware stochastic update, effectively stripping the algorithm of its spatial awareness.
4. DAR-SSA-NH (No Hybrid Update): This variant turns off the specific enhancements, specifically the Time-Varying Decay (β) and Adaptive Amplitude ($A_{i}$).

The comparative performance of these variants is presented in Table 5.

### 5.3. Parameter Sensitivity Analysis

**Figure 10:**
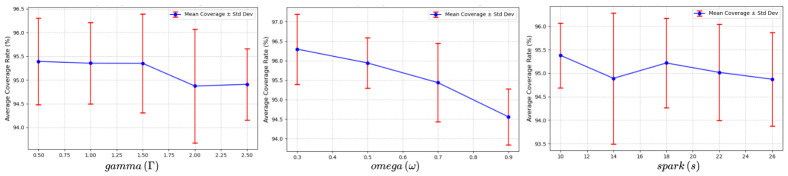
Parameter sensitivity analysis (30 independent runs).

To evaluate the stability and optimal configuration of the proposed framework, a parameter sensitivity analysis was conducted on the core variables unique to the density-aware repulsive mechanism. The analysis focused on the repulsion gain coefficient (Γ), the hybrid weighting factor (ω), and the spark count (s). Each parameter was tested across a defined range while holding the others constant, with the average coverage rate and standard deviation recorded over independent simulation runs on Experiment 1 in Table 6. The results are visualized in Figure 10. The Γ parameter controls the intensity of the spatial dispersion when nodes are clustered. It was tested across the range of [0.5, 1.0, 1.5, 2.0, 2.5]. The algorithm demonstrated strong robustness across an average coverage 95.35%, with a standard deviation of 0.9098 with Γ=0.5. The ω parameter balances the deterministic repulsive vector against stochastic exploration during the candidate generation phase. When tested across [0.3, 0.5, 0.7, 0.9], the analysis revealed a distinct peak at ω=0.3, which elevated the average coverage to 96.30% with a standard deviation of 0.9030. The spark count defines the number of local search attempts generated during the stagnation recovery phase. It was tested across [10,14,18,22,26]. The analysis showed that the spark count s=10 maintained excellent performance, with an average coverage of 95.38% and a standard deviation of 0.6893.

### 5.4. WSN Coverage Performance Optimization

We compared the proposed DAR-SSA with traditional algorithms like SSA, the classical advancement optimization model (PSO and GWO), and the two improved variants of SSA: EFSSA and EASOA to assess how well it performs in optimizing WSN node deployment while preserving a constant monitoring area and sensor types. The purpose of this comparison was to evaluate DAR-SSA’s performance in enhancing Wireless Sensor Network coverage and optimization.

#### 5.4.1. Simulation Parameter Settings

Experiment 1 evaluates a high-density scenario (small area, many nodes), simulating urban or small-scale monitoring. Experiment 2 models a low-density scenario (large area, fewer nodes), typical of remote environmental or ecological applications. To ensure a fair comparison, the optimization algorithm’s population size is fixed at 30 with a maximum of 300 iterations for all tests. Table 4 provides the complete parameter specifications.

#### 5.4.2. Coverage Experiment Result Analysis

The DAR-SSA’s performance in WSN coverage optimization is validated through two simulated experiments involving different scenarios. The algorithm was compared against three other algorithms. To ensure statistical reliability, each algorithm underwent 30 independent runs, and the average results were recorded. Table 7 confirms that DAR-SSA outperforms the comparison algorithms across all metrics in both experiments.

**Figure 11:**
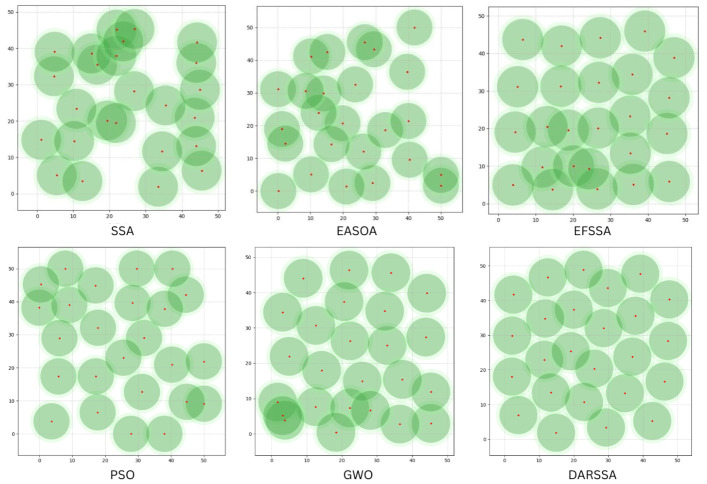
Coverage optimization comparison chart (Experiment 1).

**Figure 12:**
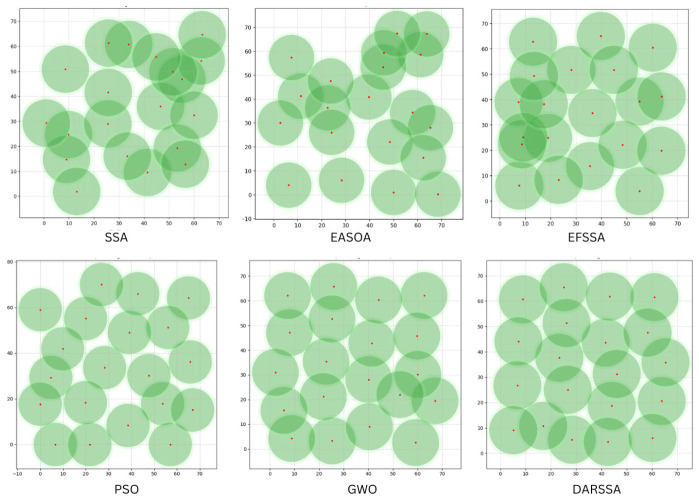
Coverage optimization comparison chart (Experiment 2).

Figure 11 and Figure 12 illustrate the resulting coverage maps. Visual inspection confirms that DAR-SSA provides superior coverage in both scenarios, exhibiting a more uniform node distribution with significantly fewer redundancies. This demonstrates the algorithm’s robustness and excellent adaptability to diverse monitoring environments.

**Figure 13:**
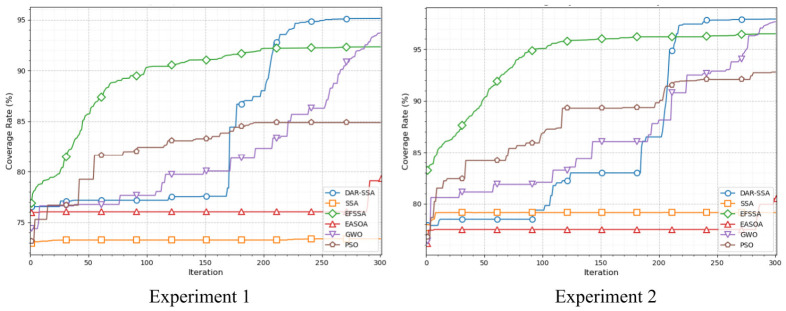
Algorithm coverage optimization convergence curves.

In each of the two experimental environments, all algorithms underwent 300 iterations, and their convergence curves are depicted in Figure 13. Notably, DAR-SSA exhibited superior optimization capabilities compared to the other five algorithms. While the other five algorithms tended to converge after 200 iterations and struggled to escape local optima, DAR-SSA continued to refine its search for a better optimum.

**Figure 14:**
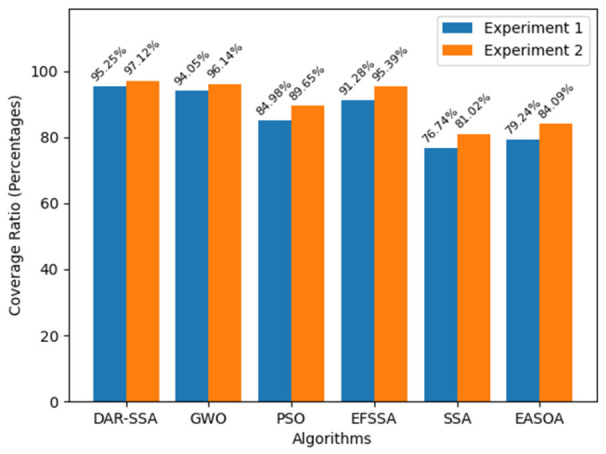
Coverage comparison in different experiments with various algorithms.

The coverage results for all algorithms in the two experimental environments are presented in Figure 14. The results indicate that DAR-SSA ultimately outperforms the other three algorithms in terms of coverage. In Experiment 1, it hit 95.25%, which is the highest in the group. And in Experiment 2, it improved further to 97.12%, again securing the top spot.

We also ran empirical tests to evaluate the actual computational overhead introduced by the density calculations and repulsive forces. Specifically, we compared the efficiency of our proposed DAR-SSA against the standard SSA using the high-density WSN setup from Experiment 1. To ensure the results were statistically reliable, we recorded the execution times across 30 independent simulation runs for each algorithm. These average runtimes are summarized in Table 8.

While incorporating local density metrics and the Soft-Core Repulsive Potential field naturally adds some computational weight to each iteration, DAR-SSA still manages to achieve a lower overall execution time than the standard SSA. This efficiency comes from the algorithm’s more intentional movement strategy. By actively pushing nodes away from dense clusters, the repulsive vector cuts down on wasted exploratory steps. Because the algorithm zeroes in on an optimal layout more directly, the time saved by avoiding unnecessary searches easily outweighs the extra time spent calculating the densities.

## 6. Limitations and Future Work

DAR-SSA demonstrates strong coverage performance across varying network scales but also exhibits important limitations. As the number of sensor nodes or the size of the coverage area increases, the computational process requires longer processing times and reduces scalability for extensive networks. This increased complexity can challenge real-time or resource-constrained applications.

Future research will focus on four key directions to address these challenges. First, our future research will aim to improve adaptability in dynamic environments, optimize energy efficiency, and validate the algorithm through real-world implementation. Second, we plan to extend the DAR-SSA framework to three-dimensional (3D) deployment scenarios, such as UAV swarms or underwater sensor networks, where the coverage optimization problem involves more complex spatial degrees of freedom. Third, we intend to incorporate a comprehensive energy consumption model that accounts for the mechanical energy required for sensor movement. This will allow the optimization objective to balance maximum coverage with minimal energy expenditure, extending the operational lifespan of WSNs. Finally, we will integrate network connectivity constraints, explicitly modeling communication radii and routing paths to ensure end-to-end data reachability to base stations.

## 7. Conclusions

This study addresses the critical challenges of coverage holes and node redundancy in Wireless Sensor Networks (WSNs) by proposing the Density-Aware Repulsive Sparrow Search Algorithm (DAR-SSA). Unlike contemporary metaheuristic variants that rely on blind Lévy flights or chaos mappings to inject untargeted diversity, the DAR-SSA framework integrates physics-based repulsive forces with a constant-spark perturbation mechanism to employ a biased stochastic, spatially aware hybrid model. While traditional heavy-tailed coordinate jumps are mathematically blind to the geometric density of the surrounding environment, often inadvertently placing nodes into coverage holes or pushing them outside the functional network boundary, the repulsive force deployed in DAR-SSA acts as a calculated directional bias. It utilizes exact local neighborhood densities to influence the underlying probabilistic search, actively steering agents away from geometric redundancies. By strictly enforcing a crowding threshold based on the hardware sensing radius, DAR-SSA effectively reduces premature convergence, leading to a much more uniform distribution of nodes. This precise action minimizes overlapping sensing radii, ensuring that every deployed node maximally contributes to expanding the global coverage footprint rather than wasting placement potential in already-monitored zones. Experimental validation across 21 standard benchmark functions and two WSN coverage scenarios, further refined by a rigorous component ablation study, demonstrates that DAR-SSA achieves superior convergence accuracy and stability compared to the standard SSA, EFSSA, EASOA, PSO and GWO, all while significantly reducing computational complexity. Specifically, in WSN coverage tasks, the finalized DAR-SSA attained the highest effective coverage rates of 95.25% and 97.12%, with statistical significance confirmed via the Wilcoxon rank-sum test. Consequently, the findings demonstrate that the repulsive mechanism and simplified recovery architecture extend beyond standard exploratory heuristics. Rather, they serve as essential, physics-informed mechanisms that maximize spatial utilization and mitigate coverage gaps, thereby providing a robust framework for large-scale node deployment. Even though calculating local densities adds a slight computational cost per iteration, this is more than offset by the algorithm’s ability to avoid redundant searches, ultimately leading to faster overall convergence.

## Figures and Tables

**Table 1:** The 21 benchmark functions.

Name	Formula	Search Range	Dim	$f_{min}$	Category
Sphere	$F_{1}=\sum_{i=1}^{n}x_{i}^{2}$	[−100, 100]	30	0	U
Schwefel 1.2	$F_{2}=\sum_{i=1}^{n}{\left(\sum_{j=1}^{i}x_{j}\right)}^{2}$	[−10, 10]	30	0	U
Rosenbrock	$F_{3}=\sum_{i=1}^{n}\left(100{\left(x_{i+1}-x_{i}^{2}\right)}^{2}+{\left(x_{i}-1\right)}^{2}\right)$	[−30, 30]	30	0	U
Step	$F_{4}=\sum_{i=1}^{n}{\left(x_{i}+0.5\right)}^{2}$	[−100, 100]	30	0	U
Exponential	$F_{5}=exp(0.5\sum_{i=1}^{n}x_{i})$	[−10, 10]	30	0	U
Sum power	$F_{6}=\sum_{i=1}^{n}{\left|x_{i}\right|}^{\left(i+1\right)}$	[−1, 1]	30	0	U
Sum square	$F_{7}=\sum_{i=1}^{n}{ix}_{i}^{2}$	[−10, 10]	30	0	U
Cigar	$F_{8}=x_{1}^{2}+10^{6}\sum_{i=2}^{n}x_{i}^{2}$	[−100, 100]	30	0	U
Zakharov	$F_{9}=\sum_{i=1}^{n}x_{i}^{2}+{\left(\sum_{i=1}^{n}{0.5ix}_{i}\right)}^{2}+{\left(\sum_{i=1}^{n}{0.5ix}_{i}\right)}^{4}$	[−5.12, 5.12]	30	0	U
Tablet	$F_{10}=10^{6}x_{1}^{2}+\sum_{i=2}^{n}x_{i}^{2}$	[−10, 10]	30	0	U
Elliptic	$F_{11}=\sum_{i=1}^{n}{\left(10^{6}\right)}^{\frac{i-1}{n-1}}x_{i}^{2}$	[−100, 100]	30	0	U
Ackley	$F_{12}=-20exp(-0.2\sqrt{\frac{1}{n}\sum_{i=1}^{n}x_{i}^{2}})-exp(\frac{1}{n}\sum_{i=1}^{n}cos({2\pi x}_{i})+20+e)$	[−32, 32]	30	0	M
Levy	$F_{13}={sin}^{2}({3\pi x}_{1})+\sum_{i=1}^{n-1}{\left(x_{i}-1\right)}^{2}[1+{sin}^{2}({3\pi x}_{i+1})]+\left|x_{n}-1\right|\cdot [1+{sin}^{2}({2\pi x}_{n})]$	[−2, 2]	30	0	M
Rastrigin	$F_{14}=\sum_{i=1}^{n}\left(x_{i}^{2}-10cos({2\pi x}_{i})+10\right)$	[−5.12, 5.12]	30	0	M
Griewank	$F_{15}=\frac{1}{4000}\sum_{i=1}^{n}x_{i}^{2}-\prod_{i=1}^{n}cos(\frac{x_{i}}{\sqrt{i}})+1$	[−600, 600]	30	0	M
Schwefel 2.26	$F_{16}=-\sum_{i=1}^{n}\left[x_{i}sin(\sqrt{\left|x_{i}\right|})\right]$	[−500, 500]	30	−418.98	M
NCRastrigin	$F_{17}=\sum_{i=1}^{n}(y_{i}^{2}-10cos({2\pi y}_{i})+10),\;$ $y_{i}=\{\begin{array}{c} x_{i},\;\;\;\;\;\;\;\;\;\;\;\;\;\;\;\;\;\;\;\;\;\;\;\;\;\;\;\left|x_{i}\right|<0.5\\ round(2x_{i})/2,\;\;\;\left|x_{i}\right|\geq 0.5 \end{array}$	[−5.12, 5.12]	30	0	M
Penalized 1	$F_{18}=\frac{\pi }{n}\left\{\sum_{i=1}^{n-1}{\left(y_{i}-1\right)}^{2}[1+10{sin}^{2}({\pi y}_{i+1})]+{\left(y_{n}-1\right)}^{2}+10{sin}^{2}({\pi y}_{i})\right\}+\sum_{i=1}^{n}u(x_{i},\;10,\;100,4)$ $y_{i}=1+\frac{x_{i}+1}{4},\;u(x_{i},\;a,\;k,m)=\{\begin{array}{c} k{\left(x_{i}-a\right)}^{m},\;{\;\;\;\;\;\;\;\;\;\;\;\;\;\;x}_{i}>a\\ 0,\;\;\;\;\;\;\;\;\;\;\;\;\;\;\;\;\;\;-a\leq x_{i}\leq a\\ k{\left({-x}_{i}-a\right)}^{m},{\;\;\;\;\;\;\;x}_{i}<-a \end{array}$	[−10, 10]	30	0	M
Weierstrass	$F_{19}=\sum_{i=1}^{n}(\sum_{k=0}^{k_{max}}\left[a^{k}cos(2\pi b^{k}(x_{i}+0.5))\right])-n\sum_{k=0}^{k_{max}}[a^{k}cos(2\pi b^{k}\cdot 0.5)],\;a=0.5,b=3,k_{max}=20$	[−1, 1]	30	0	M
Solomon	$F_{20}=1-cos(2\pi \sqrt{\sum_{i=1}^{n}x_{i}^{2}})+0.1\sqrt{\sum_{i=1}^{n}x_{i}^{2}}$	[−20, 20]	30	0	M
Bohachevsky	$F_{21}=\sum_{i=1}^{n-1}\left[x_{i}^{2}+2x_{i+1}^{2}-0.3cos({3\pi x}_{i})-0.4cos({4\pi x}_{i+1})+0.7\right]$	[−5, 5]	30	0	M

**Table 2:** Parameter settings of the comparison algorithms.

Algorithm	Title 3
SSA	st=0.8,PD=20%, SD=10%
EFSSA	$\beta_{0}=1,\;\gamma =1,PD=20\%,\;SD=15\%,\;st=0.8$
EASOA	$\beta_{initial}=0.5,\;\gamma =0.7,\;\alpha =0.5,PD=20\%,\;SD=10\%,\;st=0.8\;$
PSO	$\omega =0.7;c_{1}=2;c_{2}=2;r_{1},r_{2}=\left[0,1\right]$
GWO	$a=\left[0,2\right];r_{1},r_{2}=\left[0,1\right];C=\left[0,2\right]$
DAR-SSA	$SD=10\%,\gamma =2,\;\;\lambda =2,st=0.8,\;R_{start}=80\%,\;R_{end}=20\%,$ Γ=0.5, $A_{base}=0.2,s=10,\;\omega =0.3$

**Table 3:** Comparison results of 6 algorithms.

Functions		SSA	EFSSA	EASOA	GWO	PSO	DAR-SSA
F1	Mean	9.14 × 10^−9^	**0.00 × 10^0^**	8.59 × 10^−7^	4.26 × 10^−44^	6.94 × 10^2^	**0.00 × 10^0^**
	Std	3.60 × 10^−8^	**0.00 × 10^0^**	3.41 × 10^−6^	5.97 × 10^−44^	3.21 × 10^2^	**0.00 × 10^0^**
	Best	1.15 × 10^−199^	**0.00 × 10^0^**	2.34 × 10^−12^	1.29 × 10^−45^	3.04 × 10^2^	**0.00 × 10^0^**
	Worst	1.97 × 10^−7^	**0.00 × 10^0^**	1.90 × 10^−5^	2.29 × 10^−43^	1.64 × 10^3^	**0.00 × 10^0^**
F2	Mean	1.51 × 10^−6^	**0.00 × 10^0^**	5.42 × 10^−5^	5.25 × 10^−11^	3.45 × 10^4^	**0.00 × 10^0^**
	Std	3.13 × 10^−6^	**0.00 × 10^0^**	7.59 × 10^−5^	1.12 × 10^−10^	5.99 × 10^3^	**0.00 × 10^0^**
	Best	9.93 × 10^−171^	**0.00 × 10^0^**	2.94 × 10^−9^	6.37 × 10^−15^	2.11 × 10^4^	**0.00 × 10^0^**
	Worst	1.29 × 10^−5^	**0.00 × 10^0^**	2.75 × 10^−4^	4.42 × 10^−10^	4.57 × 10^4^	**0.00 × 10^0^**
F3	Mean	**1.79 × 10^−7^**	5.77 × 10^−5^	2.85 × 10^1^	2.65 × 10^1^	2.81 × 10^5^	1.29 × 10^−6^
	Std	**6.09 × 10^−7^**	6.76 × 10^−5^	3.17 × 10^−1^	8.68 × 10^−1^	2.00 × 10^5^	2.76 × 10^−6^
	Best	**9.47 × 10^−25^**	8.73 × 10^−8^	2.79 × 10^1^	2.52 × 10^1^	5.43 × 10^4^	4.94 × 10^−10^
	Worst	**2.75 × 10^−6^**	2.68 × 10^−4^	2.89 × 10^1^	2.87 × 10^1^	8.46 × 10^5^	1.26 × 10^−5^
F4	Mean	1.08 × 10^−8^	9.68 × 10^−8^	2.60 × 10^0^	1.59 × 10^−1^	1.04 × 10^3^	**2.28 × 10^−9^**
	Std	4.59 × 10^−8^	1.20 × 10^−7^	3.69 × 10^−1^	1.65 × 10^−1^	1.85 × 10^3^	**7.94 × 10^−9^**
	Best	**9.58 × 10^−19^**	8.91 × 10^−11^	1.65 × 10^0^	1.39 × 10^−5^	2.56 × 10^2^	1.50 × 10^−13^
	Worst	2.49 × 10^−7^	4.58 × 10^−7^	3.31 × 10^0^	5.03 × 10^−1^	1.08 × 10^4^	**4.44 × 10^−8^**
F5	Mean	**7.18 × 10^−66^**	**7.18 × 10^−66^**	1.00 × 10^−37^	2.65 × 10^1^	1.16 × 10^−58^	**7.18 × 10^−66^**
	Std	**1.05 × 10^−81^**	**1.05 × 10^−81^**	5.08 × 10^−37^	8.68 × 10^−1^	6.25 × 10^−58^	**1.05 × 10^−81^**
	Best	**7.18 × 10^−66^**	**7.18 × 10^−66^**	2.33 × 10^−52^	2.52 × 10^1^	7.18 × 10^−66^	**7.18 × 10^−66^**
	Worst	**7.18 × 10^−66^**	**7.18 × 10^−66^**	2.83 × 10^−36^	2.87 × 10^1^	3.48 × 10^−57^	**7.18 × 10^−66^**
F6	Mean	4.05 × 10^−19^	**0.00 × 10^0^**	2.54 × 10^−18^	1.21 × 10^−151^	1.23 × 10^−5^	**0.00 × 10^0^**
	Std	1.33 × 10^−18^	**0.00 × 10^0^**	1.28 × 10^−17^	5.32 × 10^−151^	2.02 × 10^−5^	**0.00 × 10^0^**
	Best	1.18 × 10^−101^	**0.00 × 10^0^**	2.56 × 10^−46^	1.78 × 10^−164^	1.58 × 10^−7^	**0.00 × 10^0^**
	Worst	6.88 × 10^−18^	**0.00 × 10^0^**	7.15 × 10^−17^	2.94 × 10^−150^	8.22 × 10^−5^	**0.00 × 10^0^**
F7	Mean	2.70 × 10^−8^	**0.00 × 10^0^**	3.83 × 10^−5^	5.65 × 10^−45^	6.64 × 10^2^	**0.00 × 10^0^**
	Std	6.58 × 10^−8^	**0.00 × 10^0^**	1.96 × 10^−4^	1.09 × 10^−44^	4.49 × 10^2^	**0.00 × 10^0^**
	Best	6.64 × 10^−168^	**0.00 × 10^0^**	1.57 × 10^−10^	5.39 × 10^−47^	4.31 × 10^1^	**0.00 × 10^0^**
	Worst	3.17 × 10^−7^	**0.00 × 10^0^**	1.09 × 10^−3^	5.81 × 10^−44^	1.74 × 10^3^	**0.00 × 10^0^**
F8	Mean	4.01 × 10^−3^	**0.00 × 10^0^**	5.88 × 10^−1^	7.75 × 10^−38^	4.98 × 10^8^	**0.00 × 10^0^**
	Std	7.31 × 10^−3^	**0.00 × 10^0^**	1.66 × 10^0^	1.26 × 10^−37^	2.35 × 10^8^	**0.00 × 10^0^**
	Best	1.63 × 10^−193^	**0.00 × 10^0^**	5.86 × 10^−7^	7.81 × 10^−40^	1.22 × 10^8^	**0.00 × 10^0^**
	Worst	3.04 × 10^−2^	**0.00 × 10^0^**	8.60 × 10^0^	5.01 × 10^−37^	1.17 × 10^9^	**0.00 × 10^0^**
F9	Mean	5.70 × 10^−10^	**0.00 × 10^0^**	5.47 × 10^−4^	3.73 × 10^−20^	1.32 × 10^2^	**0.00 × 10^0^**
	Std	1.33 × 10^−9^	**0.00 × 10^0^**	8.95 × 10^−4^	6.69 × 10^−20^	2.82 × 10^1^	**0.00 × 10^0^**
	Best	8.11 × 10^−29^	**0.00 × 10^0^**	5.74 × 10^−7^	1.75 × 10^−22^	7.20 × 10^1^	**0.00 × 10^0^**
	Worst	4.72 × 10^−9^	**0.00 × 10^0^**	3.62 × 10^−3^	2.55 × 10^−19^	2.00 × 10^2^	**0.00 × 10^0^**
F10	Mean	9.72 × 10^−9^	**0.00 × 10^0^**	1.09 × 10^−6^	1.54 × 10^−45^	4.75 × 10^2^	**0.00 × 10^0^**
	Std	3.64 × 10^−8^	**0.00 × 10^0^**	3.56 × 10^−6^	2.84 × 10^−45^	1.98 × 10^2^	**0.00 × 10^0^**
	Best	2.37 × 10^−201^	**0.00 × 10^0^**	3.00 × 10^−13^	2.77 × 10^−48^	1.05 × 10^2^	**0.00 × 10^0^**
	Worst	2.01 × 10^−7^	**0.00 × 10^0^**	2.00 × 10^−5^	1.09 × 10^−44^	8.01 × 10^2^	**0.00 × 10^0^**
F11	Mean	1.36 × 10^−3^	**0.00 × 10^0^**	1.81 × 10^−4^	1.39 × 10^−40^	5.42 × 10^7^	**0.00 × 10^0^**
	Std	3.74 × 10^−3^	**0.00 × 10^0^**	8.01 × 10^−4^	1.79 × 10^−40^	5.32 × 10^7^	**0.00 × 10^0^**
	Best	1.54 × 10^−190^	**0.00 × 10^0^**	2.49 × 10^−10^	1.52 × 10^−42^	4.66 × 10^6^	**0.00 × 10^0^**
	Worst	1.79 × 10^−2^	**0.00 × 10^0^**	4.45 × 10^−3^	6.33 × 10^−40^	2.37 × 10^8^	**0.00 × 10^0^**
F12	Mean	1.11 × 10^−4^	**4.44 × 10^−16^**	5.07 × 10^−4^	2.08 × 10^1^	2.00 × 10^1^	**4.44 × 10^−16^**
	Std	1.16 × 10^−4^	**0.00 × 10^0^**	7.71 × 10^−4^	7.33 × 10^−2^	3.12 × 10^−7^	**0.00 × 10^0^**
	Best	**4.44 × 10^−16^**	**4.44 × 10^−16^**	4.35 × 10^−6^	2.07 × 10^1^	2.00 × 10^1^	**4.44 × 10^−16^**
	Worst	3.88 × 10^−4^	**4.44 × 10^−16^**	3.31 × 10^−3^	2.10 × 10^1^	2.00 × 10^1^	**4.44 × 10^−16^**
F13	Mean	9.51 × 10^−8^	7.98 × 10^−8^	1.63 × 10^0^	6.52 × 10^−1^	5.86 × 10^−1^	**2.94 × 10^−9^**
	Std	2.46 × 10^−7^	8.99 × 10^−8^	1.25 × 10^−1^	1.52 × 10^−1^	4.21 × 10^−1^	**4.32 × 10^−9^**
	Best	**1.79 × 10^−17^**	4.99 × 10^−10^	1.36 × 10^0^	3.65 × 10^−1^	2.41 × 10^−2^	2.86 × 10^−13^
	Worst	1.15 × 10^−6^	3.67 × 10^−7^	1.84 × 10^0^	9.18 × 10^−1^	1.48 × 10^0^	**1.49 × 10^−8^**
F14	Mean	3.93 × 10^−7^	**0.00 × 10^0^**	3.50 × 10^−4^	2.34 × 10^0^	1.99 × 10^2^	**0.00 × 10^0^**
	Std	1.21 × 10^−6^	**0.00 × 10^0^**	1.16 × 10^−3^	4.62 × 10^0^	3.75 × 10^1^	**0.00 × 10^0^**
	Best	**0.00 × 10^0^**	**0.00 × 10^0^**	1.07 × 10^−10^	**0.00 × 10^0^**	9.08 × 10^1^	**0.00 × 10^0^**
	Worst	6.37 × 10^−6^	**0.00 × 10^0^**	6.00 × 10^−3^	1.82 × 10^1^	2.77 × 10^2^	**0.00 × 10^0^**
F15	Mean	1.40 × 10^−9^	**0.00 × 10^0^**	3.01 × 10^−8^	3.92 × 10^−3^	1.04 × 10^1^	**0.00 × 10^0^**
	Std	3.81 × 10^−9^	**0.00 × 10^0^**	9.66 × 10^−8^	7.22 × 10^−3^	1.69 × 10^1^	**0.00 × 10^0^**
	Best	**0.00 × 10^0^**	**0.00 × 10^0^**	3.38 × 10^−13^	0.00 × 10^0^	2.14 × 10^0^	**0.00 × 10^0^**
	Worst	1.82 × 10^−8^	**0.00 × 10^0^**	4.69 × 10^−7^	3.30 × 10^−2^	9.98 × 10^1^	**0.00 × 10^0^**
F16	Mean	**−9.71 × 10^3^**	−7.39 × 10^3^	−4.21 × 10^3^	−6.30 × 10^3^	−9.04 × 10^3^	−8.52 × 10^3^
	Std	2.92 × 10^3^	6.90 × 10^2^	5.48 × 10^2^	1.02 × 10^3^	7.54 × 10^2^	**2.74 × 10^2^**
	Best	**−1.26 × 10^4^**	−8.97 × 10^3^	−5.46 × 10^3^	−7.84 × 10^3^	−1.05 × 10^4^	−8.91 × 10^3^
	Worst	−3.82 × 10^3^	−5.83 × 10^3^	−2.84 × 10^3^	−3.60 × 10^3^	−7.56 × 10^3^	**−7.98 × 10^3^**
F17	Mean	8.03 × 10^−7^	**0.00 × 10^0^**	1.12 × 10^−4^	1.75 × 10^1^	2.16 × 10^2^	**0.00 × 10^0^**
	Std	2.69 × 10^−6^	**0.00 × 10^0^**	3.27 × 10^−4^	1.64 × 10^1^	3.09 × 10^1^	**0.00 × 10^0^**
	Best	**0.00 × 10^0^**	**0.00 × 10^0^**	5.00 × 10^−9^	0.00 × 10^0^	1.71 × 10^2^	**0.00 × 10^0^**
	Worst	1.38 × 10^−5^	**0.00 × 10^0^**	1.74 × 10^−3^	9.56 × 10^1^	2.87 × 10^2^	**0.00 × 10^0^**
F18	Mean	3.16 × 10^−9^	5.45 × 10^−9^	2.06 × 10^−1^	1.38 × 10^−2^	2.47 × 10^0^	**2.05 × 10^−11^**
	Std	6.02 × 10^−9^	6.80 × 10^−9^	3.33 × 10^−2^	8.52 × 10^−3^	1.22 × 10^0^	**2.95 × 10^−11^**
	Best	**3.80 × 10^−22^**	1.28 × 10^−10^	1.33 × 10^−1^	2.30 × 10^−6^	8.24 × 10^−1^	1.63 × 10^−15^
	Worst	2.38 × 10^−8^	2.56 × 10^−8^	2.73 × 10^−1^	2.98 × 10^−2^	5.73 × 10^0^	**1.34 × 10^−10^**
F19	Mean	**0.00 × 10^0^**	**0.00 × 10^0^**	1.14 × 10^1^	7.29 × 10^0^	**0.00 × 10^0^**	**0.00 × 10^0^**
	Std	**0.00 × 10^0^**	**0.00 × 10^0^**	1.79 × 10^0^	3.60 × 10^0^	**0.00 × 10^0^**	**0.00 × 10^0^**
	Best	**0.00 × 10^0^**	**0.00 × 10^0^**	6.98 × 10^0^	3.53 × 10^0^	**0.00 × 10^0^**	**0.00 × 10^0^**
	Worst	**0.00 × 10^0^**	**0.00 × 10^0^**	1.52 × 10^1^	2.08 × 10^1^	**0.00 × 10^0^**	**0.00 × 10^0^**
F20	Mean	6.04 × 10^−6^	**0.00 × 10^0^**	9.99 × 10^−2^	1.77 × 10^−1^	1.90 × 10^0^	6.37 × 10^−121^
	Std	1.35 × 10^−5^	**0.00 × 10^0^**	2.58 × 10^−6^	4.23 × 10^−2^	4.06 × 10^−1^	3.44 × 10^−120^
	Best	1.13 × 10^−68^	**0.00 × 10^0^**	9.99 × 10^−2^	9.99 × 10^−2^	1.30 × 10^0^	0.00 × 10^0^
	Worst	6.54 × 10^−5^	**0.00 × 10^0^**	9.99 × 10^−2^	2.00 × 10^−1^	2.70 × 10^0^	1.91 × 10^−119^
F21	Mean	1.39 × 10^−7^	**0.00 × 10^0^**	8.06 × 10^−6^	**0.00 × 10^0^**	2.56 × 10^1^	**0.00 × 10^0^**
	Std	3.48 × 10^−7^	**0.00 × 10^0^**	2.27 × 10^−5^	**0.00 × 10^0^**	1.56 × 10^1^	**0.00 × 10^0^**
	Best	1.13 × 10^−68^	**0.00 × 10^0^**	2.17 × 10^−11^	**0.00 × 10^0^**	9.89 × 10^0^	**0.00 × 10^0^**
	Worst	1.42 × 10^−6^	**0.00 × 10^0^**	1.11 × 10^−4^	**0.00 × 10^0^**	9.28 × 10^1^	**0.00 × 10^0^**
+/−/≈		2/17/2	1/4/16	0/21/0	0/20/1	0/20/1	~
Rank		3	2	6	4	5	1

**Table 4:** Wilcoxon test significant comparisons.

Comparison	Conclusion
DAR-SSA vs. SSA	DAR-SSA > SSA (*p* = 0.005618)
DAR-SSA vs. EFSSA	DAR-SSA > EFSSA (*p* = 0.023200)
DAR-SSA vs. EASOA	DAR-SSA > EASOA (*p* = 0.0000298)
DAR-SSA vs. GWO	DAR-SSA > GWO (*p* = 0.0000443)
DAR-SSA vs PSO	DAR-SSA > PSO (*p* = 0.000297)

**Table 5:** Ablation study results across WSN scenarios.

Algorithm Variant	Disable Mechanism	WSN Experiment 1 Coverage Rate
DAR-SSA-NA	Dynamic 80/20 Transition	89.34%
DAR-SSA-NR	Density-Aware Repulsion	85.47%
DAR-SSA-NH	Hybrid Candidate Generation	83.87%
Full DAR-SSA	None (Proposed Framework)	95.25%

**Table 6:** Network coverage experimental parameter settings.

Parameters	Experiment 1	Experiment 2
Area size S	50 m×50 m	70 m×70 m
Number of nodes *N*	25	20
Sensing radius $r_{s}$	6 m	10 m
Extended sensing radius $r_{e}$	0.5 m	0.5 m

**Table 7:** Network coverage experiment results.

Experiment	Criteria	SSA	EFSSA	EASOA	PSO	GWO	DAR-SSA
Experiment 1	Mean	76.74%	91.28%	79.24%	84.98%	94.05%	**95.25%**
	Std	0.0186	0.0172	0.0201	0.0211	0.0307	**0.0099**
	Max	79.50%	94.92%	83.80%	88.80%	96.37%	**97.34%**
	Min	71.32%	88.50%	74.55%	80.03%	78.22%	**93.11%**
Experiment 2	Mean	81.02%	95.39%	84.09%	89.65%	96.14%	**97.12%**
	Std	0.0206	0.0114	0.0180	0.0199	0.0436	**0.0087**
	Max	84.82%	97.72%	87.28%	93.15%	98.70%	**98.73%**
	Min	75.91%	92.80%	80.42%	85.93%	81.60%	**95.23%**

**Table 8:** Average computational execution time (30 independent runs).

Algorithm	Average Execution Time (Seconds)
Original SSA	29.9545
Proposed DAR-SSA	22.6819

## Data Availability

The original contributions presented in this study are included in the article. Further inquiries can be directed to the corresponding author.
